# Thyroid Cartilage Compression Causing Bow Hunter’s Syndrome

**DOI:** 10.1177/00034894221077477

**Published:** 2022-02-12

**Authors:** Xinyuan Hong, Emmanuel D’heygere, Eitan Prisman

**Affiliations:** 1 Division of Otolaryngology – Head & Neck Surgery, University of Ottawa, Ottawa, ON, Canada; 2 Division of Otolaryngology, Head and Neck Surgery, AZ Groeninge, Kortrijk, West-Vlaanderen, Belgium; 3 Division of Otolaryngology – Head & Neck Surgery, University of British Columbia, Vancouver, BC, Canada

**Keywords:** Bow Hunter’s syndrome, vertebral artery compression, thyroid cartilage, laryngoplasty

## Abstract

**Objectives and Methods:**

We report a unique case of Bow Hunter’s syndrome with a dominant aberrantly coursing right vertebral artery (VA), presenting with persistent dizziness and syncope despite previous decompressive surgery at vertebral levels C5-C6.

**Results:**

Re-evaluation with computed tomography-scan during provocation of dizziness by neck rotation revealed compression of the right VA at level C6 from against the ipsilateral posterior border and superior cornu of the thyroid cartilage. Laryngoplasty resulted in complete resolution of symptoms.

**Conclusion:**

This extremely rare cause of Bow’s Hunter’s syndrome should be considered, especially in refractory cases after neurosurgical decompression, and surgical management is straightforward and successful.

## Introduction

“Bow Hunter’s syndrome” (BHS), also known as rotational vertebral artery (VA) occlusion syndrome or rotational vertebrobasilar insufficiency (VBI), was coined in 1978 when a patient suffered from Wallenberg syndrome due to posterior circulation stroke during archery practice.^1^ Positional VBI occurs with VA stenosis or occlusion on head movement, often in the context of pre-existing inadequate flow in the contralateral side. There are approximately 200 cases reported in literature to date, with etiology including osteophytes, disk herniation, tendinous bands and tumors.^2^ VBI symptoms and signs include dizziness, vertigo, ataxia, syncope, paresthesia, dysarthria, vision problems and Wallenberg’s syndrome. The mechanical causes may be addressed by surgical decompression and/or C1-C2 fusion, which usually resolve symptoms.^2,3^ We report the case of a patient with persistent positional VBI, despite neurosurgical decompression, with intermittent rotational compression and occlusion of the right VA by the posterior edge of the thyroid cartilage.

## Case Report

### Presentation

**Figure 1.**
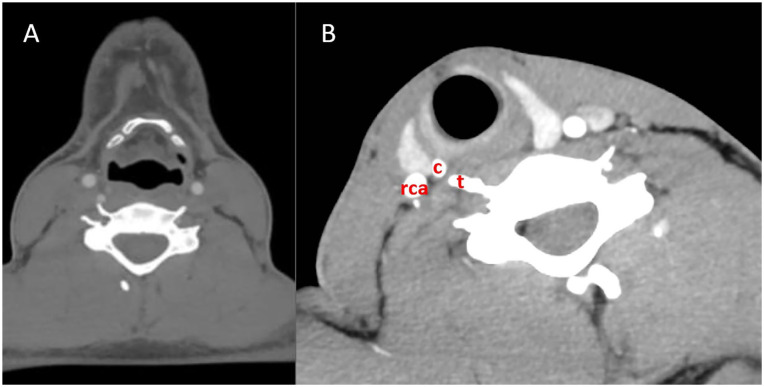
(A) Computed tomography (CT)-scan of the neck in neutral position, axial image at level C5. No obstruction is visible. Aberrant course of the right vertebral artery (VA) is evident, along with a hypoplastic left VA. (B) Computed tomography (CT)-scan of the neck with rotation of the neck to the right, axial image at level C6. The superior cornu (c) of the thyroid cartilage is seen, with the anterior tubercle (t) of C6 transverse process almost touching the thyroid cartilage. The right carotid artery (RCA) is seen, and the jugular vein is compressed. The right vertebral artery is completely occluded and is not visible on this scan at this level.

A 29-year-old male carpenter initially presented to the referring facility with a 4-year history of recurrent vertigo and syncope with right rotation and extension of the neck. Computed tomography (CT)-scan and magnetic resonance imaging (MRI)-scan 2 years prior revealed a dominant right VA, with an aberrant course. The right VA traversed anterior to an enlarged C6 tubercle of the transverse process, between longus colli and longus capitis muscles at levels C4-5, and entered the foramen transversarium at C4 (Figure 1A). The left VA was hypoplastic, and there were no significant posterior communicating arteries. Digital substraction angiography (DSA) demonstrated positional complete right VA occlusion at level C6. This was felt to be secondary to compression between the longus colli and the longus capitis muscles against an enlarged anterior C6 tubercle of the transverse process (Figure 1B). He underwent a surgical decompression of the right VA at levels C5-C6 with removal of the enlarged anterior tubercle of C6. This was performed with intraoperative catheter angiography to confirm adequate free mobilization. The procedure was complicated by temporary post-operative dysphagia, and permanent mild Horner’s syndrome. Post-operatively, while his positional VBI symptoms improved, they persisted. He was managed conservatively and counseled to avoid extreme neck rotation and low dose aspirin was prescribed to prevent stroke.

**Figure 2.**
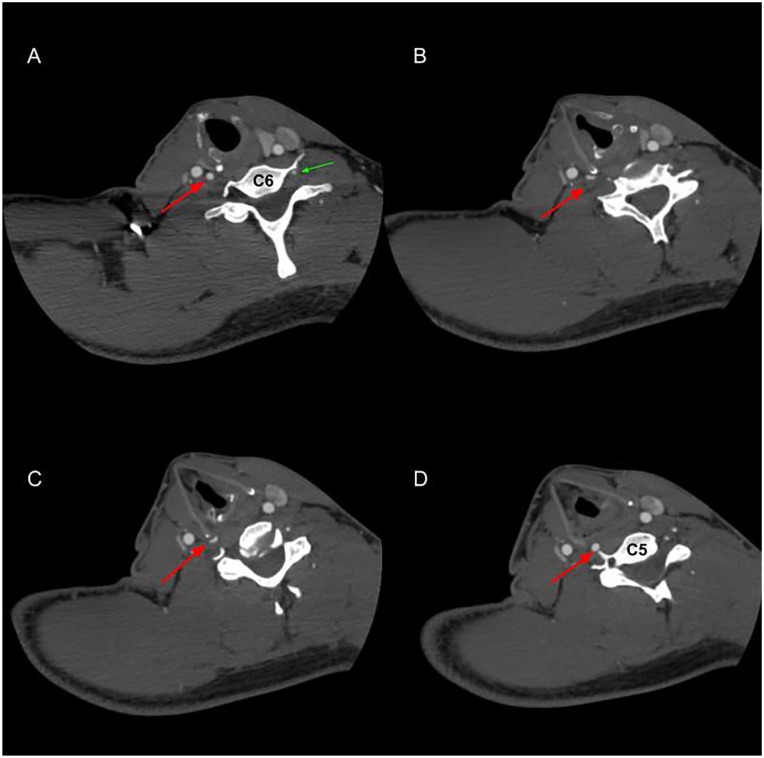
Computed tomography (CT)-scan of the neck during maximal ipsilateral rotation of the neck causing dizziness. (A-D) Axial images from caudal (level vertebra C6) to cranial (level vertebra C5). Long arrow: right vertebral artery (VA) compressed by the lateral border and superior cornu of the thyroid cartilage. Triangular arrow: left hypoplastic VA. Note lacking anterior tubercle of the transverse process C6 due to previous compressive surgery.

He was subsequently referred the Otolaryngology department, 2 years post surgical intervention, with worsening intermittent positional vertigo and syncope when turning his head to the right. Physical exam of the neck, oral cavity and flexible nasopharyngoscopy was normal, except for a mild Horner’s syndrome on the right. A new CT-scan during provocation of symptoms by right sided neck rotation revealed significant focal narrowing of the right VA at C6 level from extrinsic compression from the adjacent posterior border and superior cornu of the thyroid cartilage (Figure 2). After multidisciplinary discussion and patient consent, a new decompressive surgery was performed consisting of removing the posterior edge and superior cornu of the thyroid cartilage.

#### Surgery

**Figure 3.**
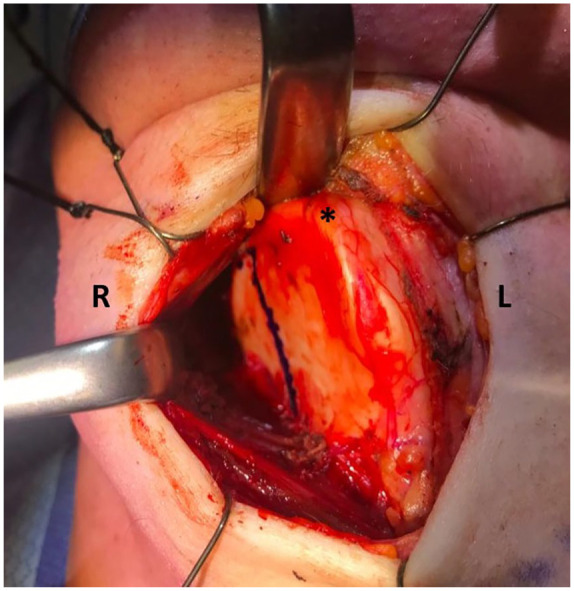
Perioperative image of the thyroid cartilage with resection marked out on the right side of the thyroid cartilage. Strap muscles are retracted anteriorly. Abbreviations: L, left; R, right; *, thyroid notch.

The procedure was done under general anesthesia. After freeing the thyroid cartilage from the overlying straps, the resection was marked out to include the lateral thyroid cartilage and superior cornu (Figure 3). The cartilage was soft and amenable to resection with a scalpel. The inner layer of thyroid perichondrium was preserved in order to protect the recurrent laryngeal nerve and laryngeal musculature. The patient was discharged home after an overnight observation. Postoperatively at 1-month follow-up, the patient was completely asymptomatic, even at extreme neck rotation. The patient has not had any further syncopal episodes to date at more than 2 years post surgery.

## Discussion

Bow Hunter’s syndrome is a rare entity involving vertebrobasilar insufficiency caused by obstruction of one or both VAs with head rotation. Symptoms include dizziness and syncopal falls and repetitive intimal injury may lead to stroke of the posterior circulation.^4^ Approximately one third of cases occur in the setting of vascular anomalies including hypoplastic, stenotic or absent contralateral VA and/or posterior communicating arteries.^2^ Most frequently, positional VA occlusion occurs at the craniocervical junction due to relative immobilization of the artery at C1-2,^5^ or in the subaxial cervical spine due to bony compression by an osteophyte within the foramen transversarium.^2^

Our case involves an exceedingly uncommon cause of BHS, due to a compression of the aberrantly coursing right VA by the posterior edge and superior cornu of the thyroid cartilage, in the context of a hypoplastic contralateral VA. The right VA entered at the C4 foramen transversarium in our patient, which usually occurs at C6 (90% at C6, 7% at C5, and 3% at C7).^4^ Only 3 other reports in the literature have noted BHS secondary to anomalous vascular entrance into the foramen transversarium and subsequent compression by the anterior structures.^6,7^ The thyroid cartilage was involved in 2 of them. In the case reported by Dabus et al,^7^ the patient was only symptomatic in extreme positions and thus was managed conservatively. In the case reported by Karle et al,^8^ occlusion of an aberrant VA by the superior cornu of the thyroid cartilage from head flexion lead to ischemic infarcts, and the patient was managed by laryngoplasty.

Computed tomography and angiography (CT/CTA), magnetic resonance image and angiography (MRI/MRA) are routinely done to evaluate any anomalies in bony structures and arteries, as well as to detect any infarcts.^9^ However, as shown in Figure 1A, this unusual cause of compression can easily be overlooked on a traditional CT-scan. Instead, a CT-scan with ipsilateral extension and rotation of the neck proved to be very helpful (Figure 2). However, as demonstrated in Figure 1B, when there is no flow at all through the VA, it is also more difficult to localize the exact cause. Therefore, ipsilateral rotation and extension should not be exaggerated. Transcranial Doppler or duplex sonography may also be used.^10,11^

Our case is unique in clearly demonstrating complete resolution of BHS symptoms post-thyroid cartilage resection. Furthermore, on the initial imaging before unsuccessful neurosurgical decompression, a prominent anterior tubercle of the C6 transverse process was visible and thought to be cause BHS. It was only after rescanning the patient because of persisting symptoms that the thyroid cartilage was identified as compressing the ipsilateral VA. This emphasizes the importance of post-operative re-evaluation and consideration of this cause primarily and especially after unsuccessful decompression. As symptoms did improve mildly after the initial surgery, compression might have been caused by both thyroid cartilage and a prominent C6 transverse process.

There are currently no guidelines on the management due to the rarity of the disease entity. Conservative medical therapies include neck immobilization and long-term antiplatelet or anticoagulation therapy. Stenosis at C1-C2 may be treated with C1-2 fusion, but this frequently causes limitations in neck rotation.^12^ Surgery consists of VA decompression. This is usually highly successful, with 87% of patients experiencing long-term symptom improvement or resolution.^2^ Of note however, among cases with residual symptoms post-decompression, 4 cases involved symptom recurrence after initial improvement.^12,13^ Kawaguchi et al reports a case with restenosis 2 months after decompression from the anterior tubercle of C6, longus colli muscle and anterior scalenus muscles. Matsuyama et al^12^ reports 3 cases of rotational VA occlusion at the C1-2 level that underwent decompression by C1 or C1-2 partial transversectomy. Restenosis occurred 2 to 3 months postoperatively, with 1 case involving a right cerebellar infarction. Rescue C1-2 posterior fusion with bone grafts was performed in 2 of the cases. Restenoses in these cases were attributed to constriction of the VA by adhesion of surrounding soft tissue.^12,13^ In our case, decompressive surgery consisting of removing the posterior edge and superior cornu of the thyroid cartilage proved to be straightforward, low-risk, and successful.

## Conclusions

We report an exceedingly uncommon case of BHS due to compression of an aberrantly coursing right dominant VA by the thyroid cartilage. The patient presented after an initially unsuccessful decompression consisting of removing the anterior tubercle of C6 transverse process. It is important to consider this cause in patients presenting with rotational VBI symptoms and especially when symptoms persist after initial decompression. This cause can be managed by laryngoplasty which proved be a straightforward and successful procedure.
